# Acceptabilité et satisfaction de l’utilisation du système *Pregnancy and Newborn Diagnostic Assessment* (PANDA) pour les soins prénatals au Burkina Faso

**DOI:** 10.11604/pamj.2020.37.361.25167

**Published:** 2020-12-21

**Authors:** Adja Mariam Ouédraogo, Rachidatou Compaoré, Anthony Somé, Désiré Lucien Dahourou, Kadari Cissé, Halima Tougri, Seni Kouanda

**Affiliations:** 1 Institut de Recherche en Sciences de la Santé (IRSS), Wemtenga, Ouagadougou, Burkina Faso,; 2 Institut Africain de Santé Publique (IASP), Ouagadougou, Burkina Faso

**Keywords:** Acceptabilité, satisfaction, mHealth, PANDA, soins prénatals, soins de santé primaires, Burkina Faso, Acceptability, satisfaction, mHealth, PANDA, antenatal care, primary health care, Burkina Faso

## Abstract

**Introduction:**

les technologies mobiles en santé sont de plus en plus utilisées comme solutions innovantes pour améliorer les services de soins prénatals (SPN) dans les soins de première ligne. Cette étude a évalué l´acceptabilité et la satisfaction de l´utilisation du système Pregnancy and Newborn Diagnostic Assessment (PANDA) lors des SPN au Burkina Faso.

**Méthodes:**

une étude transversale utilisant une approche mixte a été menée auprès de 35 utilisatrices des SPN et de 35 agents de santé dans le district sanitaire de Koupéla, dans la région du Centre-Est du Burkina Faso en septembre 2017. Des entretiens et 4 focus groups ont été réalisés auprès des utilisatrices des SPN et des d´entretiens semi-structurés auprès des professionnels de soin. Une analyse descriptive des données quantitatives a été effectuée avec le logiciel SPSS et les données qualitatives ont fait l’objet d´une analyse thématique avec NVivo 10.

**Résultats:**

le système PANDA a été très bien accepté et très apprécié par les utilisatrices et les prestataires de soins. Les motifs de satisfaction chez les utilisatrices étaient l´amélioration des échanges avec les prestataires de soin et l´accès à des soins de meilleure qualité à moindre coût. Les agents de santé ont apprécié la pertinence du système PANDA et l´amélioration des prestations fournies, du suivi et de la prise en charge des femmes enceintes.

**Conclusion:**

le système PANDA est très bien accepté et apprécié au niveau des soins primaires aussi bien par les prestataires de soins que par les utilisatrices de services de soins prénataux au Burkina Faso.

## Abstract (en)

**Introduction:**

mobile health technologies are increasingly being used as innovative solutions to improve antenatal care in Primary Care Services (PCSs). This study assessed the acceptability and satisfaction with PANDA system used in PCSs in Burkina Faso.

**Methods:**

we conducted a cross-sectional mixed-methods study of 35 users of PCSs and 35 health workers in the Koupela Health District, in the Central East region of Burkina Faso in September 2017. Interviews and 4 focus groups were conducted among PCSs users and semi-structured interviews among health-care professionals. Quantitative data analysis was carried out using the SPSS software and qualitative data analysis using a thematic analysis with NVivo 10.

**Results:**

PANDA system was very well accepted and appreciated by users and healthcare providers. Factors influencing customer satisfaction included the improvement of interactions with health care providers and the access to better quality care at lower cost. Health care providers appreciated the relevance of PANDA system as well as service improvements, follow-up and monitoring of pregnant women.

**Conclusion:**

in primary health-care system in Burkina Faso, PANDA system is very well accepted and appreciated by both health care providers and users of prenatal care services.

## Introduction

Malgré les bénéfices démontrés des soins prénatals (SPN) pour réduire la morbidité et la mortalité maternelle [1], seulement 55% des femmes enceintes réalisaient le minimum suggéré de quatre visites prénatales recommandées par l´Organisation Mondiale de la Santé (OMS) dans les pays à revenu faible ou intermédiaire (PRFI) [2]. Au Burkina Faso, des progrès ont été réalisés dans la prise en charge des femmes et des nouveau-nés, comme en témoignent les données statistiques de 2014. Le taux de naissances assistées par du personnel qualifié était de 86,2%, et le taux de fréquentation aux visites prénatales était de 84,6% pour la première consultation prénatale (CPN1), et 33,1% pour la quatrième consultation prénatale (CPN4) [3]. L´amélioration des résultats en matière de santé maternelle nécessite le renforcement des pratiques actuelles fondées sur des données factuelles. Dans cet objectif, en 2016, le Burkina Faso a expérimenté un outil de télémédecine, le système *Pregnancy And Newborn Diagnostic Assessment* (PANDA) dans le domaine de la santé maternelle et néonatale dans un district sanitaire avec l´aide de partenaires techniques et financiers.

Les téléphones mobiles en santé (mHealth) sont en effet de plus en plus utilisés dans les systèmes de soins de santé dans les PRFI et sont considérés comme des solutions innovantes offrant un potentiel immense pour améliorer les services de soins prénatals et les soins de première ligne en permettant de prendre en compte des facteurs contextuels tels que le faible niveau d´alphabétisation, les grandes distances géographiques entre les services, l´inégalité sociale, la faible qualification des ressources humaines et l´insuffisance des ressources financières [4-6]. La prolifération croissante de la technologie mobile offre de nouvelles opportunités pour permettre des soins de santé maternelle sûrs, accessibles, coordonnés et efficaces [4,7].

Le système PANDA a été conçu pour répondre aux besoins des femmes dans les contextes de rareté de ressources en matière de soins de santé pendant la grossesse, en répandant et en standardisant les soins prénatals selon les directives de l´OMS. Le système PANDA s´adresse au personnel soignant peu qualifié, appuyé à distance par des personnes qualifiées, et vise à standardiser et améliorer la qualité des visites prénatales. Des études ont montré que le système PANDA était faisable et utilisable pour effectuer des visites complètes et normalisées selon les directives de l´OMS [8,9].

Cependant, dans ces deux études, l´acceptabilité et la satisfaction au niveau des femmes et du personnel de santé n´avaient pas été étudiées. Pourtant ces deux aspects sont importants pour garantir, d´une part, la fourniture de service de qualité par les prestataires de soins, et d´autre part, l´utilisation optimale des SPN par les femmes. Cette étude avait pour objectif d´évaluer l´acceptabilité et la satisfaction des utilisateurs du système PANDA mHealth pour la fourniture de services de soins prénatals complets et normalisés en milieu rural et urbain au Burkina Faso.

## Méthodes

**Cadre de l´étude:** il s´est agi d´une étude pilote transversale descriptive dans le district sanitaire de Koupéla, dans la région du Centre-Est du Burkina Faso. Les formations sanitaires sélectionnées pour cette étude étaient le Centre de Santé et de Promotion Sociale (CSPS) urbain, le CSPS rural de Kabèga et le Centre Médical avec Antenne Chirurgical (CMA) de Koupéla. Une approche mixte concomitante a été adoptée pour cette étude.

La mise en œuvre du système PANDA à Koupéla était organisée comme suit: la collecte des données lors de la première visite prénatale par des agents de santé préalablement formés, grâce à une application à écran tactile sur des tablettes Android dans les deux CSPS. L´interface, basée sur des icônes, permet une utilisation facile et adaptable et la fourniture de soins prénataux standardisés et complets. L´application intègre une série de fonctions d´alerte pour informer les utilisateurs de résultats cliniques anormaux, de problèmes techniques ou d´informations manquantes sur le patient. Les données sont ensuite envoyées à l´hôpital de référence pour un suivi grâce à la transmission automatique entre les smartphones et l´unité médicale. Le POC est un sac contenant une plateforme de diagnostic comprenant les équipements médicaux pour l´examen physique et biologique des femmes enceintes.

L´unité médicale, système logiciel basé sur Java était hébergé au CMA de Koupéla, qui est un hôpital de référence. Toutes les informations enregistrées par l´application téléphonique étaient reçues, analysées par un personnel qualifié qui contrôlait les données et fournissait un retour d´information pertinent aux agents de santé.

**Population d´étude et échantillonnage:** la population d´étude a été constituée d´une part de l´ensemble des femmes (N=339) ayant bénéficié du système PANDA, et d´autre part de l´ensemble des agents de santé (N=35) formés à l´utilisation du système PANDA lors de la CPN ou de la validation des données de l´unité médicale. Le principal critère de sélection des participants à l´enquête qualitative a été la participation à l´étude pilote. Ainsi, trois profils de participants ont été inclus : l´utilisatrice de service CPN1 assisté de la technologie PANDA ; l´agent prestataire de soins CPN assisté de la technologie PANDA; l´administrateur impliqué dans l´étude pilote.

**Collecte des données:** les données ont été collectées par une équipe de 5 chercheurs qualifiés du 12 septembre 2016 au 10 janvier 2017. Deux focus groups (FG) de 8 à 10 femmes ayant effectué leurs CPN avec PANDA ont été réalisés dans chaque CSPS. Les utilisatrices des services CPN participantes aux FG ont été identifiées à partir du registre des femmes incluses dans l´étude pilote, au niveau des CSPS. Les FG ont eu lieu dans la communauté. La stratégie d´anonymat des participantes au cours des focus groups a consisté à leur affecter un numéro unique au début de chaque séance.

Les données des focus groups ont été collectées à l´aide d´un guide de focus group préalablement testé, portant sur l´expérience avec les mHealth, l´acceptabilité et la satisfaction. Des entretiens individuels semi-structurés ont été réalisés auprès de 13 agents de santé. Les entretiens ont porté sur l´acceptabilité et la satisfaction des agents de santé sur l´utilisation du système PANDA avec un guide d´entretien préalablement testé. Une rencontre d´échange sur le bilan de mise en œuvre des activités de l´étude pilote a également été conduite avec neuf agents de santé du CSPS urbain. Pour l´enquête quantitative, un questionnaire a été utilisé pour mesurer la satisfaction chez les utilisatrices des services CPN avec PANDA. Ce questionnaire était basé sur une échelle de satisfaction de l´utilisation de l´outil PANDA pour les CPN, allant de 1 à 10 ; la satisfaction était autoévaluée par le répondant. Le niveau 1 de l´échelle correspond à une insatisfaction totale, tandis que le niveau 10 correspond à une satisfaction totale.

**Traitement et analyse des données:** l´analyse des focus groups et des entretiens a été effectuée en plusieurs étapes. Tout d´abord, une retranscription exhaustive de toutes les entrevues a été réalisée à partir de l´enregistrement sur support numérique. Pour s’assurer de la qualité des transcriptions et éviter toute déformation des propos, elles ont été réalisées par un professionnel ayant une maîtrise parfaite de la langue locale et du français. Une analyse de contenu thématique a été effectuée avec l´appui du logiciel NVivo.10. Les données ont ensuite été dépouillées suivant un codebook décrivant les thématiques d´intérêts de l´étude, et élaborées à partir des outils de collecte et d´une lecture d´échantillon. Des analyses transversales et horizontales ont ensuite été effectuées. Pour l´analyse des données quantitatives, les données enregistrées au niveau de l´unité médicale ont été exportées sur Excel. Elles ont été analysées avec le logiciel SPSS^TM^ Statistics version 19.0. Une analyse descriptive a été effectuée. Les variables qualitatives ont été exprimées par leur fréquence, les variables quantitatives par leur moyenne. La satisfaction a été classée comme bonne pour les valeurs moyennes comprises entre 5,00 et 10,00, tandis que les valeurs inférieures à 5,00 ont été considérées comme mauvaises.

**Considérations éthiques:** l´étude a été approuvée par le Comité d´éthique pour la recherche en santé du Burkina Faso (CERS) en avril 2016 (Déclaration n°2016-04-040). Les femmes éligibles consentantes ont été invitées à participer à l´étude. Les membres du personnel impliqués dans l´étude ont signé et respecté une clause de confidentialité. Les participants avaient le droit de se retirer de l´étude à tout moment et la confidentialité et l´anonymat de toutes les femmes inscrites ont été assurés. L´étude a appliqué toutes les recommandations nationales en santé maternelle et néonatale chez toutes les bénéficiaires.

## Résultats

Au total 339 CPN1 ont été validées et analysées lors de cette phase pilote: 292 pour le CSPS urbain sur les 250 CPN1 prévues et 47 sur 50 CPN1 initialement prévues pour le CSPS de Kapèga ont été réalisées.

### Caractéristiques sociodémographiques des femmes

L´âge moyen des utilisatrices était de 26 ans (écart type: 6 ans) dans le CSPS urbain et 30 ans (écart type: 8 ans) au CSPS de Kabèga. La tranche d´âge de 19-35 ans était la plus représentée (71,7%). Elles étaient mariées dans 97,3% des cas. Environ 60,5% des enquêtées n´avaient reçu aucune instruction et seulement 25,7% de la population de l´échantillon avait le niveau d´instruction du secondaire. Le mooré était la langue la plus parlée (55,8%) (Tableau 1).

**Tableau 1:** caractéristiques socio-démographiques des femmes

	Urbain		Rural		Total	
	N	%	N	%	N	%
**Tranche d´âge**						
Moins de 19 ans	46	15,8	7	14,9	53	15,6
19-35 ans	217	74,3	26	55,3	243	71,7
Plus de 35 ans	29	9,9	14	29,8	43	12,7
**Statut matrimonial**						
Célibataire	9	3,1	0	0,0	9	2,7
Mariée/en union	283	96,9	47	100,0	330	97,3
**Existence de co-épouse**						
Oui	178	62,9	29	61,7	207	62,7
**Alphabétisée**						
Oui	121	41,4	12	25,5	133	39,2
**Niveau d´instruction**						
Aucun	167	57,2	38	80,9	205	60,5
Primaire	43	14,7	2	4,3	45	13,3
Secondaire	80	27,4	7	14,9	87	25,7
Supérieur	2	0,7	0	0,0	2	0,6
**Langue parlée**						
Français ± autre langue	124	42,5	7	14,9	131	38,6
Autre langue	15	5,1	4	8,5	19	5,6
Mooré uniquement	153	52,4	36	76,6	189	55,8

### Expérience en mHealth et PANDA

Pour les agents de santé aussi bien que pour les femmes reçues en CPN, PANDA a été leur première expérience de télémédecine et ce, quel que soit le site d´enquête. “*C´était la 1ère fois que nous voyons une chose qui s´adressait directement à nous*” (Ménagère, 39 ans, 8 enfants, focus group 2 Kabèga). “... *Moi je ne savais pas comment manipuler une tablette avant j´avais peur mais maintenant ça va*” (Entretien, Accoucheuse Auxiliaire(AA) M; CSPS urbain). Avec PANDA, les patientes ont également noté un changement dans la conduite des CPN. L´exhaustivité de l´information recueillie a suscité quelques inquiétudes au début de l´intervention sur la finalité des informations personnelles recueillies et enregistrées sur la tablette. “*Ah! Tu ne sais pas si c´est pour avoir des informations et revenir t´arrêter plus tard donc nous répondions aux questions avec méfiance! Parce que dès que tu parles on ne peut plus effacer*” (étudiante, mariée, 18 ans, primipare, FG1 CSPS urbain). Ces doutes n´avaient pas été mentionnés par les femmes du groupe 2.

### Acceptabilité du système PANDA

Pour les utilisatrices de services CPN, PANDA est “*une bonne chose*”. Comparativement à la CPN ordinaire, les femmes rencontrées dans les 4 focus groups ont toutes marqué leur préférence pour la CPN assistée avec PANDA. Ce constat a été confirmé par les agents de santé : “*les femmes adhèrent facilement d´être en tout cas enregistrées, donc jamais on n´a pas enregistré, on n´a pas rencontré une femme qui refuse en tout cas de faire (la CPN) avec PANDA*” (Entretien, AA k ; CSPS urbain). Le caractère novateur de l´outil a même plutôt suscité un intérêt à l´adhésion des femmes à l´étude pilote. “*Nous étions plutôt contentes parce qu´ils ont dit que nous sommes le 1^er^groupe à l´essai donc nous étions contentes de pouvoir être les 1^ers^à en bénéficier*” (Ménagère, 30 ans, 6 enfants, FG1 Kabèga). De même, pour les agents de santé, PANDA est une innovation pertinente. “*Cette question de PANDA en fait il faut dire que le PANDA il est révolutionnaire, révolutionnaire en ce sens que c´est un système qui va nous permettre de faire des CPN complètes*” (Entretien, médecin; CMA).

### Satisfaction des utilisatrices

Toutes les femmes rencontrées au cours des 4 focus group ont indiqué un niveau de satisfaction élevé pour la CPN avec PANDA. En effet, la médiane des notes de satisfaction des femmes lors de l´utilisation du système PANDA était de 10 avec des extrêmes de 5 et 10. Le Tableau 2 fait la synthèse des notes obtenues par PANDA lors des Focus groups. Les agents de santé ont également tous exprimé une grande satisfaction par rapport au système PANDA. “*Très satisfait malgré la charge de travail que ça entraîne. Je dis charge de travail parce que c´est de la qualité, mais la qualité aussi a un prix*.”(Entretien Médecin, CMA). “*Notre satisfaction de PANDA nous pouvons l´estimer autour de 90%”*(Agent de santé KB, CSPS urbain).

**Tableau 2:** satisfaction des femmes sur l´utilisation du système PANDA pour les soins prénataux

	Médiane	Minimum	Maximum
CSPS urbain			
Groupe 1 (n=9)	10,00	5,00	10,00
Groupe 2 (n=8)	10,00	5,00	10,00
**CSPS urbain**			
Groupe 1 (n=8)	10,00	5,00	10,00
Groupe 2 (n=10)	7,00	5,00	10,00
Ensemble (n=35)	10,00	5,00	10,00

### Motifs de satisfaction

Les motifs de satisfaction de la CPN avec PANDA étaient nombreux et variaient selon qu´il s´agissait de clientes CPN ou de prestataires de soins. Trois principaux points de satisfaction ont été dégagés des entretiens de groupe avec les clientes en CPN avec PANDA. Le premier point de satisfaction couramment évoqué était la qualité des échanges entre prestataires de soins et femmes. “*C´est grâce à la tablette que nous avons pu causer comme ça. Avant il n´y avait pas d´échange entre les agents de santé et nous. C´est la CPN avec la tablette qui a développé cette relation entre les agents de santé et nous*” (FG 1, CSPS Kabèga). Ce rapprochement a été possible grâce au repositionnement physique lors de la consultation (côte à côte au lieu de face à face), occasionné par PANDA.

“(...) *Avant tu ne pouvais pas t´approcher de l´agent de santé. La patiente est assise d´un côté et l´agent de santé est assis de l´autre côté. Avant l´agent de santé pouvait te poser une question, mais par peur tu ne répondais pas. Tu ne levais même pas la tête de peur de croiser son regard ou un visage serré. Mais maintenant tu es assise à côté de l´agent de santé et donc tu peux poser toutes les questions et donner toutes les réponses que tu veux parce qu´il y a une confiance entre vous*” (Ménagère, 36 ans, 4 enfants, FG2 urbain). Cette satisfaction a été aussi constatée chez les prestataires qui ont rapporté une augmentation du temps d´écoute des clientes et l´amélioration du climat de confiance comme des éléments de satisfaction dans l´utilisation de PANDA. “*Le temps d´écoute également pour certaines clientes qui est prolongé ça également c´est une bonne chose parce que ça met en confiance ces clientes-là, voilà, et également ça rassure ces clientes*” (Attaché de santé, ECD).

Le second point de satisfaction des femmes était relatif à la nature et à la qualité des informations et aux conseils donnés pour le suivi médical de la grossesse, le régime alimentaire et la préparation à l´accouchement. “*On m´a montré dès que tu es enceinte comment tu vas commencer à faire les pesées. Ils nous ont donné des conseils sur ce que nous devons amener au CSPS pour notre accouchement (...). Et puis le temps que tu vas tenir la grossesse jusqu´à ton accouchement, les fruits que tu dois manger. Ils ont dit qu´une femme enceinte doit dormir sous une moustiquaire*” (élève, 18 ans, primipare, Focus group 1 CSPS urbain). “*(...) C´est visible et puis c´est facile de comprendre. Sinon avant il y a des choses qu´on nous disait. Mais nous ne pouvions pas comprendre parce que ce n´était pas sur photos, ni sur image. Mais avec la tablette nous comprenions mieux donc moi je préfère la CPN avec la tablette*” (n°4, 39 ans, ménagère, 3 enfants, focus group 2 CSPS de Kabèga).

Le troisième point de satisfaction des femmes portait sur la disponibilité des analyses biologiques pratiquées sur place au CSPS, gratuitement et avec des résultats disponibles instantanément. “*Oui c´est bien parce que après les examens de sang, on sait si tu es malade ou pas, si tu es anémiée ou pas. Ils disent tout ça (...) Avec la tablette ils font les examens sans prendre de l´argent, sinon avant il fallait dépenser 2000fr pour faire les examens donc c´est vraiment bien*” (focus group 1 CSPS urbain).

Les prestataires de soins et les membres de l´équipe cadre de district (ECD) étaient également satisfaits du système PANDA. Trois principaux points de satisfactions ont été dégagés de leur expérience avec PANDA. Tout comme les utilisatrices, les agents de santé étaient également satisfaits de la possibilité d´effectuer les examens para cliniques au CSPS lors de la CPN. En effet, la plupart des examens effectués avec PANDA n´étaient pas toujours demandés dans la CPN classique. D´ailleurs, en dehors des sites d´expérimentation de PANDA, ces examens n´étaient pas tous disponibles dans les formations sanitaires périphériques. “*Il y a l´accès à plusieurs examens dont le taux d´hémoglobine et puis VDRL (test de détection de la syphilis) ça c´était jusqu´à Koupèla [35 km] qu´on pouvait avoir ça*” (AA SM, CSPS de Kabèga).

De plus, au niveau des agents de santé, la disponibilité immédiate des résultats d´examens complémentaires facilitait la prise en charge de la femme. La systématisation et le respect des différentes étapes chronologiques de la consultation conformément aux recommandations nationales en matière de soins prénataux, ainsi que la convivialité de l´interface de la tablette ont été également appréciés. Cela permettait à la fois d´améliorer la qualité des soins et des données. “*Avec PANDA le format est déjà là! ...ici tant que ce n´est pas renseigné, ça ne vous permet pas d´avancer. Donc on est obligé de tout renseigner, de collecter toutes les informations pour que la CPN soit de qualité*” (Entretien, IDE, Responsable du CSPS urbain). La validation des données au niveau du CMA, était un autre motif de satisfaction à la fois pour l´ECD et certains agents de santé du CSPS car elle permettait d´améliorer la référence et le suivi des femmes : ”*Nous avons la chance de suivre certaines femmes qui posent des problèmes parce que lors de la validation le responsable arrive à détecter telle femme qui pose problème. Et l´avantage du dépistage au niveau de PANDA des risques permet d´anticiper déjà au niveau de l´accouchement pour certaines femmes comme par exemple l´éclampsie ou l´HTA à risque pour qu´elles soient référées au niveau du CMA pour une meilleure prise en charge*” (Entretien Médecin, ECD).

## Discussion

Les résultats de la présente étude montrent que le système PANDA a été très bien accepté dans le district sanitaire de Koupéla au Burkina Faso par les agents de santé et les utilisatrices des services de santé bien qu´ils soient très peu exposés à la technologie mobile, et malgré quelques inquiétudes et méfiance au début de l´intervention. La satisfaction était très élevée auprès des utilisatrices des CPN dans le district pilote, surtout du fait de l´amélioration des échanges avec les prestataires de soin et de l´accès à des soins de meilleure qualité à moindre coût. Du côté des agents de santé, le système PANDA est apprécié parce qu´il permet l´amélioration de la qualité des soins grâce au renforcement des compétences des agents des soins de santé primaires, par l´amélioration des prestations fournies, du suivi et de la prise en charge des femmes enceintes. Par ailleurs, le recueil de données complètes et standardisées améliore le système d´information sanitaire.

L´acceptabilité du système PANDA dans cette étude confirme la réponse aux besoins d´amélioration de la qualité des soins aussi bien chez les femmes enceintes que par les prestataires de soins dans les contextes de rareté de ressources en matière de soins de santé. De plus, l´acceptabilité est également due au fait que l'application PANDA facilite la compréhension par les femmes enceintes et la manipulation par des agents de santé peu qualifiés, sans diminuer la qualité de la collecte de données [8,9]. L´acceptation des applications mhealth est souvent un véritable goulot d´étranglement auquel se heurtent tous les praticiens du changement technologique ainsi que de nombreux chercheurs. PANDA offre ainsi une solution prometteuse pour accroître l´accès aux soins prénatals de haute qualité et normalisés pour les femmes enceintes des régions isolées, comme confirmé par une étude de PANDA réalisée à Madagascar [8].

Cela est en ligne avec d´autres études montrant que les applications de téléphonie mobile pour la santé reproductive sont bien acceptées par les femmes et les agents de santé [10-12]. Le niveau de satisfaction globale des soins fournis grâce à l´application du système PANDA était très élevé chez les utilisatrices de service de soins prénataux, mais aussi chez les prestataires de soins. Cette satisfaction, chez les utilisatrices des CPN, est le résultat de la qualité des échanges prestataire-femmes, des informations et des conseils illustrés et la disponibilité des examens paracliniques gratuitement.

L´expérimentation du système PANDA a été accompagnée d´un renforcement de plusieurs composantes de qualité de soins prénataux, telles que la promotion de santé en soins maternels, néonatals et infantiles, le renforcement de la compétence des professionnels, des services offerts et de l´organisation des soins. Ainsi, le changement de technique lors de l´éducation à la santé des femmes au cours des CPN a favorisé la qualité des échanges entre prestataires et utilisatrices des CPN en réduisant la distance lors des consultations, brisant les barrières et générant un climat de confiance et accru l´attention des femmes sur les conseils fournis. Malgré le taux de satisfaction globale élevé, il convient de porter une attention particulière aux items pour lesquels les femmes enceintes ne sont pas complètement satisfaites. La plupart des commentaires ouverts sur les points d´insatisfactions par les utilisatrices étaient liés au temps d´attente. Des études sur les mhealth utilisées chez les femmes enceintes lors des CPN ont trouvé un niveau de satisfaction élevé [10-12]. Néanmoins, le niveau élevé de satisfaction doit être interprété avec précaution, car une réponse positive dans une étude de satisfaction ne veut pas toujours dire que la prise en charge était bonne mais simplement que rien de grave ne s´est produit [13].

L´étude a également révélé que les agents de santé étaient aussi très satisfaits de l´application du système PANDA. La validation des données collectées et échangées offre une opportunité de supervision à distance qui s´avère particulièrement importante car la pénurie d´agents de santé qualifiés dans les pays à faibles ressources pourrait s´aggraver à l´avenir, et nécessiter le recours à une délégation des tâches à du personnel de santé moins qualifié [14]. La supervision à distance pourrait garantir la qualité des soins et des données collectées et renforcer la capacité des agents de santé de première ligne mais aussi des agents de santé communautaires. Le contexte socioculturel des femmes pourrait constituer un biais dans cette étude. En effet, la santé retrouvée ou le fait de bénéficier d´une consultation constituait déjà pour certaines femmes une source de satisfaction et influençaient les réponses aux questions. Cela était perceptible chez les patients provenant des zones rurales. Cependant la triangulation des données semble montrer que le système PANDA est bien accepté et très apprécié au Burkina Faso.

## Conclusion

Cette étude pilote indique que le système PANDA est très bien accepté et apprécié au niveau des soins primaires aussi bien par les prestataires de soins que par les utilisatrices de services de soins prénataux au Burkina Faso. Les résultats suggèrent que cette innovation technologique représente un potentiel d´amélioration de la qualité des soins prénataux dans les contextes à ressources limitées. Cependant, malgré les premiers résultats encourageants, des études supplémentaires sont nécessaires pour évaluer l´impact du système PANDA pour la santé maternelle avant la mise à l´échelle nationale.

### Etat des connaissances sur le sujet

- Les téléphones mobiles en santé (mHealth) sont en effet de plus en plus utilisés dans les systèmes de soins de santé dans les PRFI;
- Les mHealths sont considérés comme des solutions innovantes offrant un potentiel immense pour améliorer les services de soins prénatals et les soins de première ligne;
- Des études ont montré que le système PANDA était faisable et utilisable pour effectuer des visites complètes et normalisées selon les directives de l´OMS.

### Contribution de notre étude à la connaissance

- Cette étude pilote indique que le système PANDA est très bien accepté pour des soins prénataux standardisés et de qualité au niveau des soins primaires;
- Le niveau de satisfaction est très élevé aussi bien chez les prestataires de soins que chez les utilisatrices de services de soins prénataux au Burkina Faso.

## Remerciements

Nous remercions l’ONG Enfants du monde (EdM, l´Hôpitaux Universitaires de Genève (HUG), Fondation pour le Développement Burkina Faso (FDC/BF) et la Direction de la santé de la famille du Burkina Faso qui ont permis en partie la réalisation de cette étude. Nous tenons également à exprimer nos remerciements à tous les agents de santé du district sanitaire de Koupéla qui ont, par leur encouragement et leur disponibilité, facilité la collecte des données.

## References

[1] Abalos E, Chamillard M, Diaz V, Tuncalp O, Gülmezoglu A (2016). Antenatal care for healthy pregnant women: a mapping of interventions from existing guidelines to inform the development of new WHO guidance on antenatal care. BJOG Int J Obstet Gynaecol.

[2] WHO (2016). World health statistics: monitoring health for the SDGs, sustainable development goals. WHO.

[3] Ministère de la Santé (2015). Annuaire statistique santé 2014. Ministère de la Santé. Ouagadougou.

[4] Al-Shorbaji N (2013). The world health assembly resolutions on eHealth: eHealth in support of universal health coverage. Methods Inf Med.

[5] Eysenbach G, Group C-E (2011). CONSORT-EHEALTH: improving and standardizing evaluation reports of Web-based and mobile health interventions. J Med Internet Res.

[6] Tilahun B, Smillie K, Bardosh KL, Murray M, Fitzgerald M, Cook V (2018). Identifying Barriers and Facilitators of 13 mHealth Projects in North America and Africa: Protocol for a 5-Year Implementation Science Study. JMIR Res Protoc.

[7] World Health Organization (2012). World health statistics 2012.

[8] Benski AC, Stancanelli G, Scaringella S, Herinainasolo JL, Jinoro J, Vassilakos P (2016). Usability and feasibility of a mobile health system to provide comprehensive antenatal care in low-income countries: PANDA mHealth pilot study in Madagascar. J Telemed Telecare.

[9] Borsari L, Stancanelli G, Guarenti L, Grandi T, Leotta S, Barcellini L (2018). An Innovative Mobile Health System to Improve and Standardize Antenatal Care Among Underserved Communities: A Feasibility Study in an Italian Hosting Center for Asylum Seekers. J Immigr Minor Health.

[10] Cormick G, Kim NA, Rodgers A, Gibbons L, Buekens PM, Belizán JM (2012). Interest of pregnant women in the use of SMS (short message service) text messages for the improvement of perinatal and postnatal care. Reprod Health.

[11] Jareethum R, Titapant V, Bn CT, Bn SV, Chuenwattana P, Bn JC (2008). Satisfaction of Healthy Pregnant Women Receiving Short Message Service via Mobile Phone for Prenatal Support: A Randomized Controlled Trial.

[12] Lund S, Nielsen BB, Hemed M, Boas IM, Said A, Said K (2014). Mobile phones improve antenatal care attendance in Zanzibar: a cluster randomized controlled trial. BMC Pregnancy Childbirth.

[13] Sitzia J, Wood N (1997). Patient satisfaction: a review of issues and concepts. Soc Sci Med.

[14] Organisation mondiale de la Santé (2012). Recommandations de l´OMS: optimisation des rôles du personnel de santé par la délégation des tâches pour améliorer l´accès aux interventions de santé maternelle et néonatale.

